# Analysis of glutamine synthetase target‐site mutations and their role in endowing glufosinate‐ammonium resistance

**DOI:** 10.1002/ps.70827

**Published:** 2026-05-11

**Authors:** Aimone Porri, Susee Sudhakar, Matheus M Noguera, Michael Betz, Jens Lerchl, Franck E Dayan, Ingo Meiners, Tino Caldarelli, Jason Norsworthy

**Affiliations:** ^1^ Global Research & Development Agricultural Solutions, BASF SE Ludwigshafen Germany; ^2^ Department of Crop, Soil and Environmental Sciences University of Arkansas Fayetteville AR USA; ^3^ Department of Research Centers Montana State University Huntley MT USA; ^4^ Department of Agricultural Biology Colorado State University Fort Collins Colorado USA; ^5^ BASF Agricultural Solutions US LLC Research Triangle Park North Carolina USA

**Keywords:** glufosinate resistance, TSR, mutation, palmer amaranth, *Amaranthus palmeri*, goosegrass, *Eleusine indica*

## Abstract

**BACKGROUND:**

Glufosinate‐ammonium (GFA) is a non‐selective herbicide that inhibits glutamine synthetase (GS), a key enzyme in plant nitrogen metabolism. Although resistance to GFA has been reported in several weed species, confirmed cases of target‐site resistance (TSR) *via* GS mutations remain rare. In *Amaranthus palmeri*, resistance is usually associated with GS gene amplification and overexpression, while the role of GS point mutations remains unclear. This study investigated GFA resistance in a population lacking GS amplification, and evaluated the functional relevance of putative resistance‐conferring GS mutations.

**RESULTS:**

Digital PCR revealed no substantial GS amplification or overexpression in the CCR population. Sequencing identified a glycine‐to‐aspartic acid substitution at position 255 (G255D) in the chloroplastic GS2.2 isoform, detected in ~80% of CCR plants in heterozygous form. Notably, this mutation was exclusively in untreated plants. *In vitro* assays showed that GS2.2 G255D retained ~58% of wild‐type catalytic activity and was completely insensitive to GFA inhibition. However, ectopic expression of G255D, as well as the homologous *Arabidopsis* GS2.2 G254D substitution, did not confer GFA tolerance in *Arabidopsis*. Analysis of GS1.1 variants demonstrated that mutations disrupting GFA binding generally caused severe reductions in catalytic efficiency. Re‐evaluation of the previously reported *Eleusine indica* GS1.1 S59G mutation revealed no meaningful shift in GFA sensitivity.

**CONCLUSIONS:**

GFA resistance in the CCR population cannot be explained by GS amplification or GS2.2 G255D. These findings underscore the strong structural and functional constraints on GS target‐site evolution and suggests that non‐target‐site mechanisms likely underlie GFA resistance in this population. © 2026 BASF SE and The Author(s). *Pest Management Science* published by John Wiley & Sons Ltd on behalf of Society of Chemical Industry.

## Graphical abstract

GS2.2 G255D mutation conferred enzyme‐level glufosinate insensitivity but no whole‐plant resistance, showing trade‐offs between catalytic function and herbicide binding and providing a baseline for future studies on target‐site glufosinate resistance.

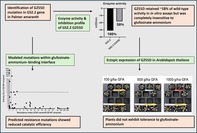

## 1 INTRODUCTION

Glufosinate‐ammonium (GFA) is a non‐selective, postemergence herbicide widely utilized for broad‐spectrum control of weeds. It targets glutamine synthetase (GS; EC 6.3.1.2), a key enzyme in nitrogen metabolism that catalyzes the ATP‐dependent condensation of glutamate and ammonia to form glutamine.^1^ In higher plants, GS exists at two different locations: GS1 in the cytosol and GS2 in the chloroplast.^2^ GS2 typically dominates foliar tissues of C3 species due to its role in photorespiratory ammonia reassimilation, while both GS1 and GS2 contribute to nitrogen processing in C4 species such as Palmer amaranth (*Amaranthus palmeri* S. Watson), where GS1 activity may account for a large proportion of total GS activity.^3^, ^4^ Inhibition of GS leads to toxic ammonia accumulation and disrupted photorespiration.^5^ Though ammonia accumulation was initially considered the primary cause of glufosinate‐induced cell death, recent studies indicate that reactive oxygen species (ROS) generation and membrane lipid peroxidation contribute greatly to its phytotoxic effects.^6^

Commercialized in the 1990s, glufosinate use expanded following the introduction of transgenic crops carrying *bar* or *pat*, which encode phosphinothricin acetyltransferase to detoxify glufosinate by converting it to *N*‐acetyl‐glufosinate.^7^ This trait is now incorporated into multiple cropping systems, including LibertyLink®, Enlist™, and XtendFlex®. In the United States, GFA applications in soybean [*Glycine max* (L.) Merr.] increased from approximately 1.08 million kg in 2012 to 6.54 million kg in 2020, highlighting its growing role in managing herbicide‐resistant weeds.^8^

Evolution of GFA resistance in weeds has been relatively limited compared to other herbicide sites of action, with only five reported GFA‐resistant species: goosegrass [*Eleusine indica* (L.) Gaertn.], Italian ryegrass [*Lolium perenne* L. ssp. *multiflorum* (Lam.) Husnot], perennial ryegrass (*Lolium perenne* L.), rigid ryegrass (*Lolium rigidum* Gaudin), and *A. palmeri*.^9^, ^10^, ^11^, ^12^, ^13^, ^14^, ^15^, ^16^ Among these, *A. palmeri* is the only broadleaf species with confirmed resistance. As a dioecious, highly fecund C4 weed with extensive gene flow and rapid adaptation capacity, *A. palmeri* presents a substantial threat to sustainable chemical control. Resistance to GFA in *A. palmeri* has been confirmed in populations from Arkansas, Missouri, and North Carolina, including accessions with multiple herbicide resistances.^16^, ^17^, ^18^, ^19^

Mechanisms of herbicide resistance in weeds are broadly classified into target‐site resistance (TSR) and non‐target‐site resistance (NTSR). TSR mechanisms include point mutations in the herbicide target gene, gene amplification, or overexpression of the target protein, all of which reduce herbicide binding efficacy or increase the availability of the target. NTSR mechanisms encompass lower absorption or translocation, vacuolar sequestration, or enhanced metabolic degradation, often mediated by cytochrome P450 monooxygenases (P450s), glutathione‐S‐transferases (GSTs), or other detoxification pathways.^20^ Glufosinate uptake can vary depending on the herbicide applied in combination, with certain mixtures reducing absorption in species such as *A. palmeri* and barnyardgrass [*Echinochloa. crus‐galli* (L.) P. Beauv.].^21^ In *A. palmeri*, the most validated mechanism of glufosinate resistance to date involves GS2 overexpression and gene amplification, with limited evidence supporting a role for target‐site mutations.^17^, ^22^

A few GS mutations have been reported or engineered in relation to glufosinate sensitivity, but none have been conclusively linked to field‐evolved resistance. A *GS2* Asp173Asn substitution in *L. multiflorum* was initially thought to confer resistance but was later shown to be non‐functional; resistance in that population was ultimately determined to be mediated by enhanced metabolism.^23^ A S59G substitution in the GS1 isoform of *E. indica* conferred glufosinate resistance, with transgenic rice (*Oryza sativa* L.) plants expressing the mutant gene exhibiting reduced sensitivity to the herbicide.^24^ In controlled systems, substitutions such as H249Y and R295K in soybean and rice GS2 enzymes have been engineered to reduce glufosinate binding, but such mutations have not been observed in weed species under field selection.^25^, ^26^

Recent research has shown that extrachromosomal circular DNA (eccDNA) is a novel mechanism underlying gene amplification in certain herbicide‐resistant weeds, such as in glyphosate‐resistant *A. palmeri*.^27^ Similarly, in a GFA‐resistant *A. palmeri* population from Arkansas (MSR2), GS2.1 and GS2.2 isoforms were co‐amplified on an eccDNA structure.^28^ This finding suggests that eccDNA‐mediated gene amplification may serve as a flexible and rapid adaptation strategy in *A. palmeri*, allowing high‐copy gene expression outside the constraints of chromosomal regulation. However, the presence of GS2‐containing eccDNA has not been confirmed in all resistant populations, and resistance mechanisms are likely to be heterogeneous across regions.

The present study investigates the response of an intriguing *A. palmeri* population from Arkansas (hereby named CCR) to GFA. In a previous study, this population displayed resistance indices of 5.1 and 5.9 compared with susceptible standards.^16^ Plants in this population lack GS2 amplification but harbor a glycine‐to‐aspartic acid substitution at position 255 (G255D), located in a conserved region of the enzyme. The mutation was detected only in heterozygous form in untreated plants, but was not found in surviving plants, raising questions about its functionality and potential role in GFA resistance. In addition, given the limited number of validated resistance‐endowing GS mutations, we conducted a modeling‐guided mutational analysis to identify GS1.1 residues involved in glufosinate binding and oligomer stability. A panel of GS1.1 variants was then generated to assess the effects of amino acid substitutions on catalytic activity and glufosinate sensitivity, defining structural constraints on GS‐mediated target‐site resistance and providing a framework for interpreting future GS variants. The objectives of this study were to: (1) characterize GS copy number, expression, and sequence variation in CCR; (2) functionally assess the enzymatic activity and glufosinate sensitivity of the GS2.2 G255D substitution identified in this population; (3) evaluate a broader panel of GS1.1 target‐site variants for their effects on catalytic efficiency and GFA inhibition; and (4) re‐examine the previously reported *Eleusine indica* GS1.1 S59G mutation using purified recombinant enzyme to clarify its contribution to GFA resistance.

## 2 MATERIALS AND METHODS

### 2.1 Plant material

The *A. palmeri* CCR population and the susceptible reference were grown in a greenhouse under a 14:10 h light: dark photoperiod. Natural light was supplemented with high‐pressure sodium lamps providing a photon flux density of approximately 1100 μmol m^−2^ s^−1^ during daylight hours. Day/night temperatures were maintained at 30/25 °C, respectively. Twenty‐three untreated CCR plants were collected for molecular characterization. In addition, another set of CCR plants was grown and treated with GFA at the labeled rate (656 g ha^−1^) when they reached 7–10 cm in height. Two weeks after application, tissue samples were collected from 15 surviving plants and submitted for molecular characterization of resistance.

### 2.2 Preparation of DNA

Leaf samples of 0.5 cm^2^ each were transferred into a sample tube (Collection microtubes; Qiagen, Hilden, Germany). Subsequently, the samples were homogenized in a shaker mill (TissueLyser II; Qiagen, Hilden, Germany) with steel beads. The process of DNA extraction was carried out in a KingFisher Flex Magnetic Particle Processor (Thermo Fisher Scientific, Schwerte, Germany), employing the Chemagic Plant 400 kit (Perkin Elmer, Rodgau, Germany), in accordance with the manufacturer's instructions (with modifications implemented by IDENTXX GmbH).

### 2.3 Preparation of total RNA

Frozen leaf samples (0.5 cm^2^ each) were transferred into a sample tube. Subsequently, the samples were homogenized with steel beads in a shaker mill (TissueLyser II; Qiagen, Hilden, Germany). The total RNA extraction was carried out in the KingFisher Flex Magnetic Particle Processor (Thermo Fisher Scientific, Schwerte, Germany), employing the MagMAX(™) Plant RNA Isolation Kit (Applied Biosystems, Darmstadt, Germany), in accordance with the manufacturer's instructions.

### 2.4 Preparation of cDNA

The random hexamer‐primed cDNA was obtained by reverse transcription of 200 ng μL^−1^ of total RNA using the PrimeScript RT Reagent Kit with gDNA Eraser (Takara Bio Europe, St‐Germain‐en‐Laye, France) according to the manufacturer's instructions.

### 2.5 GS copy number and expression analysis

The digital PCR (dPCR) was performed in a final volume of 12 μL using 1.48 μL of DNA or cDNA, 0.48 μL (0.2 μM) of specific primers and 0.24 μL (0.2 μM) of probe (Table S1) (biomers.net GmbH, Ulm, Germany), 3 μL of QIAcuity HighMultiplex ProbePCR Kit (Qiagen, Hilden Germany) and 4.64 μL PCR‐Grade H_2_O for the quintuple dPCR. dPCR was performed in a dPCR thermal cycler (QIAcuity One, 5plex Device, Qiagen, Hilden Germany) in a 96‐well nanoplate (QIAcuity Nanoplate 8.5 k 96‐well) under the following conditions: 2 min at 95 °C and 55 cycles of 15 s denaturation at 95 °C; 55 s annealing and elongation at 60 °C. The following conditions were met during the imaging of the partitions. The FAM and HEX experiments were conducted with an exposure time of 500 milliseconds, with the gain set to 6. The ROX experiment utilized an exposure time of 300 milliseconds, also with the gain set to 4. The TAMRA experiment utilized an exposure time of 400 milliseconds, also with the gain set to 6. The Cy5 experiment utilized an exposure time of 400 milliseconds, also with the gain set to 8. The assessment of copy number variation and expression analysis was conducted by employing Qiagen's QIAcuity Software Suite, version 3.1.0.0. This process entailed the utilization of positive, negative, and no‐template control wells (NTC), along with the determination of sample thresholds.

### 2.6 Identification of target‐site mutations in *A. palmeri* CCR population GS isoforms *via* cDNA sequencing

The amplification of the coding sequences was performed in a final volume of 25 μL using 5 μL of the random hexamer primed cDNA and 1 μL (10 pmol) of specific primers (Table S2), 12.5 of MyFi™ DNA Polymerase (Bioline GmbH, Luckenwalde, Germany) and 6.5 μL PCR‐Grade H2O. DNA was amplified in a PCR thermal cycler (T100 PCR thermal cycler, Bio‐Rad Laboratories GmbH, Germany) under the following conditions: 3 min at 95 °C and 35 cycles of 30 s denaturation at 95 °C; 30 s annealing at primer specific temperature and 2 min elongation at 72 °C; and a final elongation step at 72 °C for 5 min. Aliquots were taken and analyzed on 1.5% agarose gels. The PCR products were sequenced from both sites *via* Sanger sequencing (SeqLab‐Microsynth, Göttingen, Germany). Chromatograms were processed in Geneious v. 9.1.8 (Biomatters, Auckland, New Zealand), and sequences were aligned against wild‐type GS coding sequences. Resulting nucleotide substitutions were translated into amino acid changes to identify potential target‐site mutations.

### 2.7 Protein expression and purification

The full‐length of GS wild‐type and variants CDS sequences form *A. palmeri* and *E. indica* were synthesized *de novo* and cloned into the pRSetB expression vector (Invitrogen, Carlsbad, CA, USA) using BamHI and HindIII restriction sites. An N‐terminal hexahistidine tag was included to facilitate purification. Recombinant constructs were transformed into *Escherichia coli* strain BL21(DE3)pLysS (Novagen, EMD Millipore, Billerica, MA, USA), and transformants were selected on LB agar containing 100 μg mL^−1^ ampicillin and 34 μg mL^−1^ chloramphenicol.

For protein expression, a single colony was used to inoculate 3 mL LB medium with antibiotics and incubated at 37°C with shaking (200 rpm) for 6 h. A 20‐μL aliquot of this pre‐culture was transferred into 20 mL fresh LB medium and incubated overnight. The next day, 100 μL of the overnight culture was inoculated into 100 mL ZYM‐5052 autoinduction medium (see Supplemental Data for preparation), supplemented with antibiotics, and incubated at 37 °C for 5 h, followed by 21 h at 25 °C.

Cells were harvested by centrifugation at 6000×*g* for 30 min at 4 °C. Cell pellets were resuspended in PPO lysis buffer [10 mL g^−1^ pellet; 50 mM NaH₂PO₄, 100 mM NaCl, 5 mM imidazole, 5% (v/v) glycerol, pH 7.5, 20 mg mL^−1^ lysozyme, 30 U mL^−1^ DNase I] supplemented with protease inhibitors (complete EDTA‐free; Roche Diagnostics, Mannheim, Germany). Suspensions were sonicated on ice (3 min, 90% amplitude, 30‐s intervals). Cell debris was removed by centrifugation at 38000×*g* for 30 min at 4 °C, and 2 mL of 5 M NaCl was added to the clarified supernatant.

For purification, a 500‐μL bed volume of HisPur Ni‐NTA resin (Thermo Fisher Scientific, IL, USA) was equilibrated with buffer (20 mM NaH₂PO₄, 50 mM NaCl, 5 mM imidazole, 5 mM MgCl₂, 17% glycerol, 0.1 mM EDTA, pH 8.0). The supernatant was passed through the resin, washed with 5.6 mL wash buffer (20 mM NaH₂PO₄, 50 mM NaCl, 5 mM imidazole, 17% glycerol, pH 7.5), and the bound protein was eluted with 1 mL elution buffer (20 mM NaH₂PO₄, 50 mM NaCl, 250 mM imidazole, 17% glycerol, pH 7.5). Protein concentrations were determined using a Scandrop nanovolume spectrophotometer (Analytikjena, Life Science, Germany). Purity and solubility were assessed by SDS‐PAGE (10%) using 2.5 μg protein per lane.

### 2.8 Glutamine synthetase enzyme activity

GS activity was determined spectrophotometrically following the Sigma‐Aldrich enzymatic assay protocol. The reaction couples the ATP‐dependent conversion of l‐glutamate and ammonium to l‐glutamine with pyruvate kinase (PK) and l‐lactate dehydrogenase (LDH), such that oxidation of β‐NADH to β‐NAD^+^ can be monitored at 340 nm. Recombinant GS from *A. palmeri* was expressed in *E. coli*, purified by affinity chromatography, and quantified by the Bradford method using bovine serum albumin as standard. Assays were conducted at 37 °C in 3 mL total volume containing 34.1 mM imidazole (pH 7.1), 102 mM sodium l‐glutamate, 8.5 mM ATP, 1.1 mM phosphoenolpyruvate, 60 mM MgCl₂, 18.9 mM KCl, 45 mM NH₄Cl, 0.25 mM β‐NADH, 28 U PK, 40 U LDH, and 0.4–0.8 U recombinant GS. Reaction mixtures were equilibrated to 37°C and initiated by the addition of enzyme, and the decrease in absorbance at 340 nm was recorded for 5–10 min; only the linear portion of the curve was used for activity calculations. For inhibition assays, GFA was added directly to the reaction mixture to final concentrations ranging from 10^−8^ M to 10^−2^ M, using 2.5‐fold serial dilutions, with a no‐inhibitor control included in each run. Activity was calculated using an extinction coefficient for NADH of 6.22 mM^−1^ cm^−1^ and expressed as units per mg protein, where one unit corresponds to the formation of 1 μmol of l‐glutamine in 15 min at pH 7.1 and 37 °C. Background activity was corrected by including blank assays without GS. Percent inhibition was calculated relative to untreated (positive) and no‐enzyme (negative) controls. Assays were conducted in triplicates. IC₅₀ values (concentration of inhibitor reducing GS activity by 50%) were estimated by nonlinear regression (three‐ or four‐parameter log‐logistic models). Resistance factors were calculated by dividing the IC₅₀ of each mutant variant by that of wild‐type GS.

### 2.9 Transgene preparation and Arabidopsis transformation

Mutant GS variants G2.2 G255D from *A. palmeri* and G254D from *Arabidopsis* were cloned into the RTP6557 transformation vector, which was subsequently introduced into *Agrobacterium tumefaciens* strain C58C1pMP90. The construct also carried an acetolactate synthase (ALS) herbicide‐resistance gene as a selectable marker, ensuring that *Arabidopsis* seedlings tested for resistance to GFA herbicide expressed the transgene.

Agrobacterium cultures were initiated 1 d prior to transformation by inoculating 1 mL of glycerol stock into 250 mL of YEB medium (1 g L^−1^ yeast extract, 5 g L^−1^ beef extract, 5 g L^−1^ peptone, 5 g L^−1^ sucrose, and 0.49 g L^−1^ MgSO₄·7H₂O) supplemented with the appropriate antibiotic. Cultures were grown for 12 h at 28 °C with agitation at 150 rpm. On the following day, the culture density was adjusted to OD₆₀₀ = 1.0 in YEB medium, harvested by centrifugation (1600 g, 10 min), and resuspended in 150 mL of infiltration medium (2.2 g L^−1^ Murashige & Skoog salts, 50 g L^−1^ sucrose, 0.5 g L^−1^ MES hydrate, and 10 μL L^−1^ BAP [1 mg mL^−1^ stock solution]). The pH of the infiltration medium was adjusted to 5.7–5.8.

*Arabidopsis* plants carrying immature floral buds were transformed by the floral‐dip method, immersing inflorescences for 10 s in the bacterial suspension supplemented with Silwet‐L77 at 0.05% v/v. After dipping, plants were maintained overnight under high humidity and low light, then transferred to long‐day conditions until maturity. Once siliques had yellowed, seeds were harvested into paper bags. T₁ seeds were collected, placed into Falcon tubes, and stored at 4 °C.

After ≥14 d of cold storage, T₁ seeds were sown and stratified as previously described. Transgenic plants were confirmed by PCR detection of the AHAS resistance marker gene. Plants were grown under short‐day conditions for 12–14 d until the four‐leaf stage, at which point resistant individuals were transplanted into 6 × 6 cm pots containing GS90 soil. Plants were grown for an additional 10 d, then shifted to long‐day growth conditions 1 d prior to herbicide application.

Herbicide treatments were applied at the 10‐leaf stage using a spray chamber calibrated to deliver 375 L ha^−1^ of spray solution. GFA herbicide was applied at rates of 100, 500, and 1000 g active ingredient ha^−1^, with DASH HC (BASF, ref. ID no. 30059102; 349 g L^−1^ oil [fatty acid esters] and 209 g L^−1^ alkoxylated alcohols‐phosphate esters) as an adjuvant. Control plants received spray solution containing adjuvant only. Herbicide efficacy was visually evaluated 7 d after application.

### 2.10 Molecular modeling

A homology model of AMAPA cytosolic GS1.1 was built to identify residues for targeted experiments on GFA binding, with emphasis on protein–protein interactions that can influence herbicide sensitivity. The maize GS1.1 crystal structure co‐crystallized with ADP and methionine sulfoximine phosphate (PDB 2D3A served as the template.^29^ Model construction and placement of GFA into the site corresponding to methionine sulfoximine phosphate were performed in MOE using default settings. [*Molecular Operating Environment (MOE), 2024.06; Chemical Computing Group ULC, 1010 Sherbrooke St. West, Suite #910, Montreal, QC, Canada, H3A 2R7, 2024.]* For the template region resolved in the protein crystal structure (residues C3–P356), AMAPA cytosolic GS1.1 shares 88.1% sequence identity and 95.5% similarity with maize; residues directly contacting GFA are fully conserved, indicating preserved active site geometry and identical ligand interactions. However, sensitivity or resistance to GFA is also influenced by the stability and architecture of subunit interfaces in the GS oligomer. One principal interface is mainly formed by N‐terminal residues (R34–E69; Table 3), and a second interface involves a central sequence segment.

To evaluate how amino acid substitutions might affect these interfaces, interfacial residues were mapped from the homology model and a multiple sequence alignment, and prioritized candidate mutations based on evolutionary conservation and single nucleotide polymorphisms (SNPs). Selected substitutions were modeled *in silico* and assessed by molecular modeling to estimate their effects on interface stabilization. Analyses focused on potential changes to intermolecular contacts critical for oligomer integrity — notably polar and charge assisted hydrogen bonds (*e.g*. E69 to R316) and hydrophobic patches (*e.g*. V161, A163) that could alter oligomer packing or ligand access. These structure‐based considerations provide a rationale for selecting residues for experimental validation to dissect how active site binding and oligomeric interfaces jointly determine GFA sensitivity and resistance.

## 3 RESULTS

### 3.1 Copy number variation and mRNA abundance of GS isoforms in the *A* *. palmeri* CCR population

**Figure 1:**
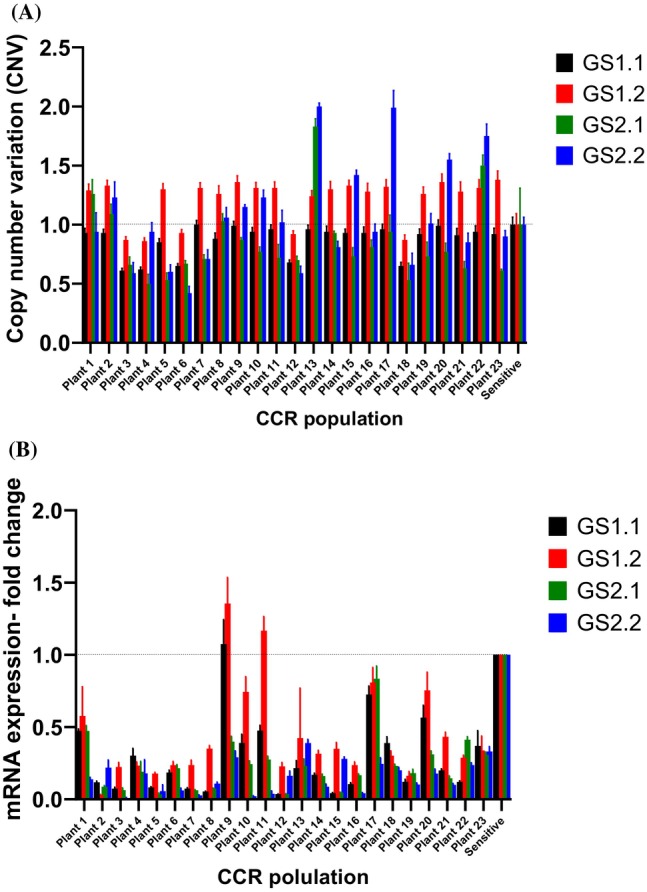
Copy number variation (CNV) (A) and mRNA abundance (B) of *Amaranthus palmeri* GS in CCR plants determined by dPCR. CNV values and expression fold change are shown for GS1.1 (black), GS1.2 (red), GS2.1 (green), and GS2.2 (blue). Values were calculated using the ΔΔC_t_ method with a known GFA‐sensitive *A. palmeri* plant as reference which contains only one copy of each GS isoforms. Error bars indicate confidence interval at 95% significance.

Digital PCR analysis was employed to detect differences in gene copy number and mRNA abundance among the four GS isoforms in 23 untreated CCR plants, using an herbicide‐sensitive plant as reference (fold change = 1.0). For the majority of CCR plants, copy number values for all isoforms were close to reference, generally falling between 0.8 and 1.5, indicating no substantial gene amplification (Fig. 1(A)). Few CCR plants exhibited slight deviations. CCR13, CCR17, and CCR22 showed a slight increase in GS2.2 CN, with fold changes reaching up to 2.0‐fold. In CCR13 and CCR22 plants, a mild elevation in copies was observed also for GS2.1, suggesting that amplification in these isoforms is not widespread but can occur in certain CCR individuals. No plant demonstrated extreme amplification (>10‐fold) for any GS isoform. Expression analysis also did not detect major target upregulation with most of the plants expressing the GS isoforms similar to or slightly lower than the sensitive plant (Fig. 1(B)). Taken together, the vast majority of CCR plants maintain GS copy numbers or expression similar to the sensitive control, with increases in GS2.1 and GS2.2 CN being found in a few individuals.

### 3.2 GS cDNA sequencing in CCR plants

The sequencing of the GS2.2 isoform in the 23 CCR untreated plants revealed a non‐synonymous substitution at position 255, where glycine (G) in the susceptible reference sequence was replaced by aspartic acid (D). This G255D substitution was present in heterozygous form in 18 of 23 plants (~80% frequency) (Table 1). This residue lies within a highly conserved domain of GS 2.2, near E192 (in GS1.1) or E251 (in GS2.1) (Fig. S1), a residue involved in GFA binding, suggesting that the amino acid substitution may have functional significance in inhibitor and/or substrate binding.^16^ Sequencing of the remaining three GS isoforms (GS1.1, GS1.2 and GS2.1) did not reveal any amino acid substitutions compared to the susceptible reference (Table 1). Given the conservation of the G255 site across GS isoforms, and the proximity with E251, the G255D substitution may alter enzyme properties and affect GFA binding to the target enzyme.

**Table 1:** GS isoforms cDNA sequencing in CCR population. Sanger sequencing was employed to detect sequence polymorphisms in 23 untreated CCR plants in all four GS isoforms

CCR population	GS1.1	GS1.2	GS2.1	GS2.2
Plant 1	NP	NP	NP	G255D+/−
Plant 2	NP	NP	NP	G255
Plant 3	NP	NP	NP	G255
Plant 4	NP	NP	NP	G255D+/−
Plant 5	NP	NP	NP	G255D+/−
Plant 6	NP	NP	NP	G255D+/−
Plant 7	NP	NP	NP	G255D+/−
Plant 8	NP	NP	NP	G255D+/−
Plant 9	NP	NP	NP	G255D+/−
Plant 10	NP	NP	NP	G255D+/−
Plant 11	NP	NP	NP	G255D+/−
Plant 12	NP	NP	NP	G255D+/−
Plant 13	NP	NP	NP	G255D+/−
Plant 14	NP	NP	NP	G255D+/−
Plant 15	NP	NP	NP	G255D+/−
Plant 16	NP	NP	NP	G255D+/−
Plant 17	NP	NP	NP	G255D+/−
Plant 18	NP	NP	NP	G255
Plant 19	NP	NP	NP	G255
Plant 20	NP	NP	NP	G255
Plant 21	NP	NP	NP	G255D+/−
Plant 22	NP	NP	NP	G255D+/−
Plant 23	NP	NP	NP	G255D+/−

NP=No polymorphisms detected and G255D+/− indicates that the substitution was heterozygous. G255 indicates that the plant retained the wild‐type allele.

### 3.3 Enzyme activity and inhibition profile of GS2.2 G255D

**Figure 2:**
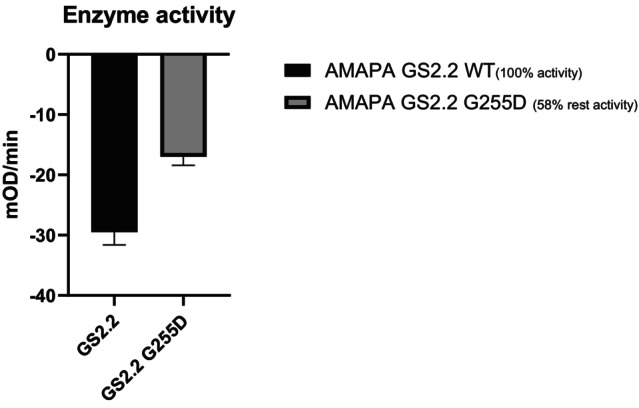
Enzymatic activity of wild‐type GS2.2 and the G255D mutant. Activities were measured *in vitro* and are expressed as the change in absorbance per min (mOD min^−1^). Wild‐type GS2.2 activity was set to 100% (black bar), while the G255D variant retained 58% of WT activity (grey bar). Bars represent mean values ± SE of three independent enzyme preparations (*n* = 3). Statistical significance was assessed using a two‐tailed Student's *t*‐test (*P* < 0.05).

**Figure 3:**
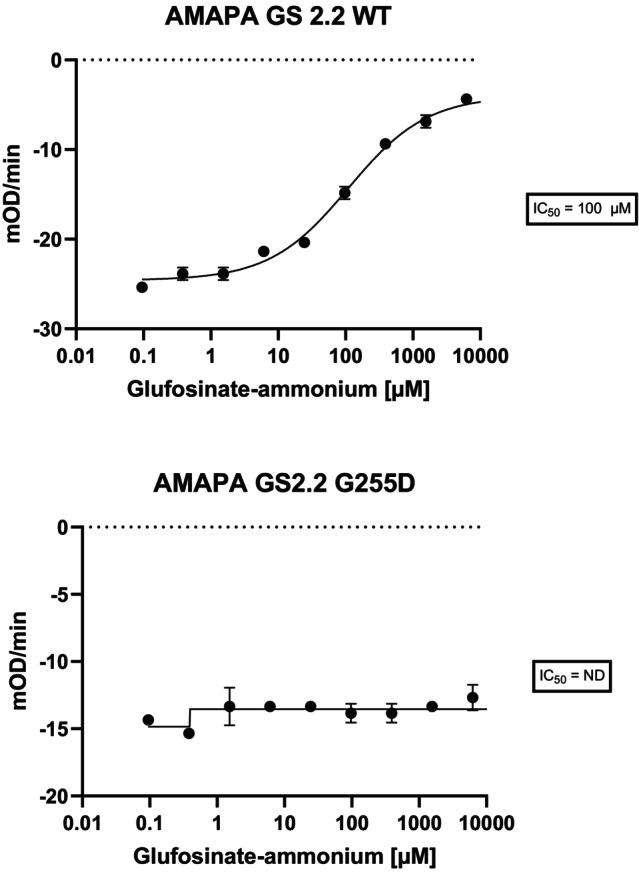
Inhibition of *Amaranthus palmeri* GS2.2 wild‐type (WT) and G255D mutant enzymes by GFA. Enzyme activity was measured as the change in absorbance per min (mOD min^−1^) across a range of GFA concentrations. The WT GS2.2 enzyme (upper panel) exhibited a sigmoidal dose–response curve with an IC₅₀ of 100 μM. The G255D mutant (lower panel) showed no detectable inhibition within the tested concentration range, and an IC₅₀ could not be determined (ND). Data points represent the mean ± standard error of three replicate measurements.

Enzyme assays comparing the wild‐type GS2.2 and the G255D variant revealed a substantial reduction in catalytic activity associated with the amino acid substitution. The wild‐type GS 2.2 displayed full activity, measured at approximately −30 mOD min^−1^ (Fig. 2). In contrast, the G255D variant retained only 58% of wild‐type activity, corresponding to approximately −17 mOD min^−1^. This lower activity indicates that the G255 residue plays a role in substrate interaction and/or catalytic efficiency. The data supports the hypothesis that the G255D substitution, located in a highly conserved domain, alters GS2.2 enzyme function. In addition, enzyme inhibition assays demonstrated a striking difference in sensitivity to GFA between the wild‐type GS2.2 and the G255D variant (Fig. 3). The wild‐type enzyme displayed a typical dose–response curve, with activity decreasing as GFA concentration increased, resulting in an IC₅₀ of 1.1 × 10^−4^ M. In contrast, the GS2.2 G255D variant maintained stable activity across the full range of GFA concentrations tested, with no measurable inhibition and an IC₅₀ value that could not be determined. This absence of inhibition indicates that the G255D substitution confers a high level of resistance to GFA *in vitro*, consistent with the residue predicted role in substrate binding and inhibitor interaction.

### 3.4 Ectopic expression of G255D in transgenic *Arabidopsis*

**Figure 4:**
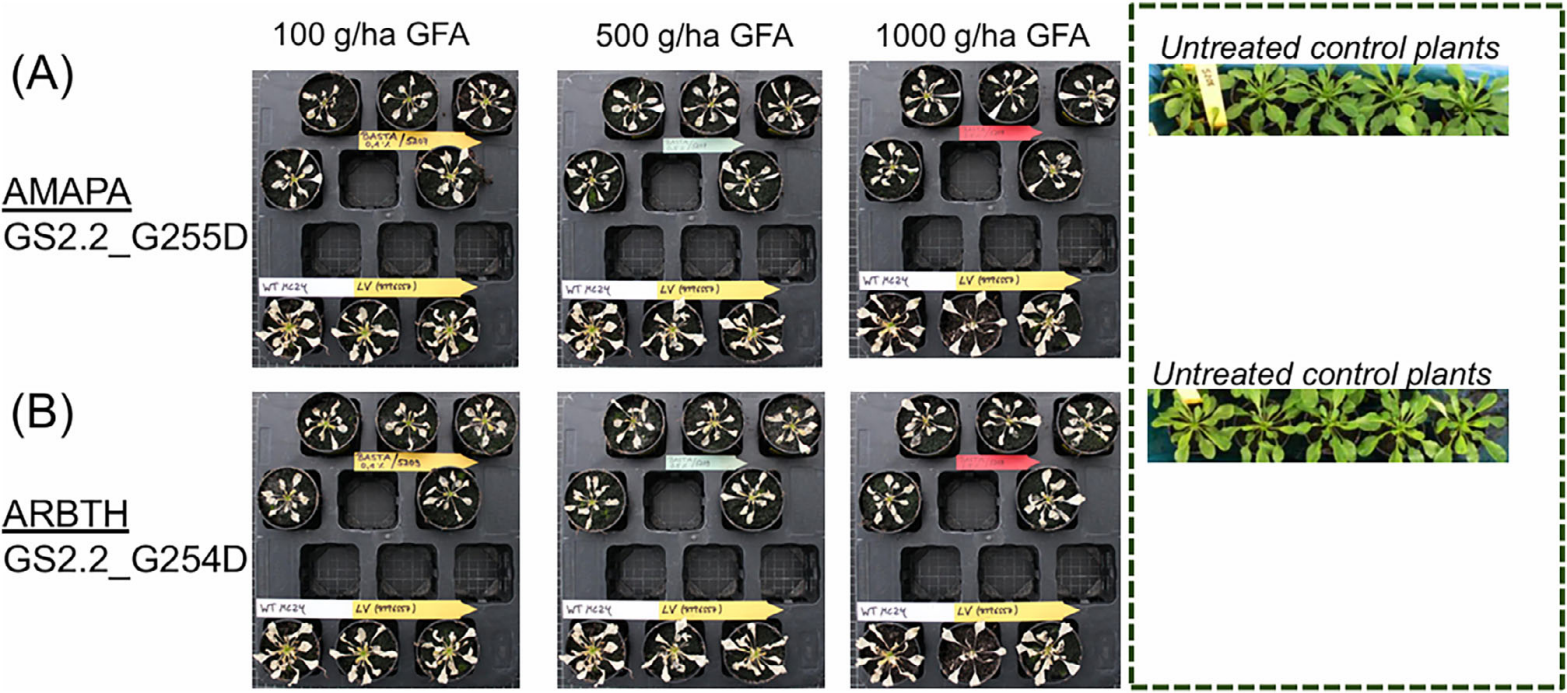
Response of (A) *Arabidopsis thaliana* plants expressing *Amaranthus palmeri* GS2.2 G255D or G254D to postemergence GFA application. Independent T₁ transgenic events expressing GS2.2_G255D A or G254D (B) (upper five pots in each tray) were sprayed at the rosette stage with GFA at 100, 500, or 1000 g a.i. ha^−1^. Wild‐type plants (WT MC24) and empty vector controls (LV RTP6557) are shown in the bottom row of each tray. Photographs were taken 10 days after treatment. Transgenic plants were confirmed by PCR detection of the AHAS resistance marker gene. Untreated control plants are also shown.

Transgenic plants ectopically expressing the GS2.2 G255D variant were evaluated for tolerance to GFA at three application rates (100, 500, and 1000 g ha^−1^). Across all concentrations, plants expressing G255D showed chlorosis and necrosis comparable to wild‐type controls, with no improvement in injury or survival (Fig. 4(A)). Despite conferring strong resistance to inhibition by GFA *in vitro*, the G255D substitution alone is insufficient to provide GFA tolerance in planta under the conditions tested. In addition, G255D was ectopically expressed using the *Arabidopsis* GS2.2 backbone (G255D of *A. palmeri* corresponds to G254D in *A. thaliana*). Like G255D, G254D failed to confer tolerance to GFA in the transgenic plants (Fig. 4(B)).

### 3.5 GS2.2 sequencing in GFA survivors

Because the GS2.2 G255D substitution was initially identified exclusively in untreated CCR plants collected for molecular characterization, it remained unclear whether this variant might also be present in survivors of GFA applications. To address this possibility, another batch of CCR plants was grown and sprayed with a labeled rate of GFA (656 g ha^−1^) at the 7–10 cm stage. Two weeks after treatment, 15 survivors were tissue sampled and submitted to sequencing of the GS2.2 coding region as previously described. Surprisingly, none of the survivors carried the G255D substitution (Table S4). When considered together with the enzyme inhibition assays, which showed complete insensitivity of the G255D variant to GFA but reduced catalytic activity, and the *Arabidopsis* transgenic experiments, in which ectopic expression of G255D failed to confer GFA tolerance in planta, these results indicate that G255D does not contribute to GFA resistance at the whole‐plant level in the CCR population.

### 3.6 GS1 target‐site mutations effect on GFA sensitivity

A set of cytosolic GS1.1 variants from *A. palmeri* was assayed for sensitivity to GFA. These variants were generated on residues important for GFA binding^16^ (Fig. S1). Variants were created by a single nucleotide change in the codon encoding for each selected residue (Table S3). The wild‐type enzyme (WT) was strongly inhibited by GFA, with an IC₅₀ of 22.5 μM and complete inhibition (100%) at 10 mM inhibitor, consistent with high susceptibility.

In contrast, many GS1.1 variants harboring substitutions at residues E131, E192, G245, H249, R291, R311, or R332 displayed extreme loss of sensitivity, with IC₅₀ values exceeding 10 mM. At saturating inhibitor concentrations, inhibition rarely exceeded 10%, but residual activities were typically <2% of WT (Table 2). Several variants (*e.g*. E192G, G245N/R/V, H249Q/P/Y, R311G, R332C/H/K) were catalytically inactive, precluding IC₅₀ estimation. The highest remaining activity observed was for the G245C variant (1.8%), while all the others were < 1% relative to the wild‐type enzyme.

**Table 2:** Enzymatic activity and glufosinate‐ammonium sensitivity of cytosolic GS1.1 variants from *Amaranthus palmeri*

GS1.1 Isoform	Mutation	Glufosinate‐ammonium IC_50_ [μM] *P* < 0.0001	Glufosinate‐ammonium % Inhibition at saturation 10 000 μM	Remaining activity [%]
Cytosolic	**WT**	**22.5**	**100**	**100**
Cytosolic	E131A	>10 000	8.5	0.04
Cytosolic	E131D	>10 000	48	0.2
Cytosolic	E131G	>10 000	7.2	0.04
Cytosolic	E131K	>10 000	0	0.04
Cytosolic	E131Q	>10 000	6.5	0.04
Cytosolic	E131V	>10 000	6.6	0.03
Cytosolic	E192A	Enzyme inactive	‐	‐
Cytosolic	E192D	>10 000	0	0.89
Cytosolic	E192G	Enzyme inactive	‐	‐
Cytosolic	E192K	>10 000	1.3	0.03
Cytosolic	E192Q	>10 000	0	0.03
Cytosolic	E192V	Enzyme inactive	‐	‐
Cytosolic	G245A	Enzyme inactive	‐	‐
Cytosolic	G245C	>10 000	0	1.81
Cytosolic	G245S	>10 000	9	0.2
Cytosolic	G245N	Enzyme inactive	‐	0
Cytosolic	G245R	Enzyme inactive	‐	0
Cytosolic	G245V	Enzyme inactive	‐	0
Cytosolic	H249L	>10 000	7.8	0.03
Cytosolic	H249N	>10 000	17	0.06
Cytosolic	H249Q	Enzyme inactive	‐	‐
Cytosolic	H249P	Enzyme inactive	‐	‐
Cytosolic	H249Y	Enzyme inactive	‐	‐
Cytosolic	H249R	>10 000	29	0.02
Cytosolic	R291C	>10 000	0	0.07
Cytosolic	R291S	>10 000	0.3	0.08
Cytosolic	R291G	>10 000	0	0.06
Cytosolic	R291H	>10 000	0	0.1
Cytosolic	R291L	>10 000	0	0.05
Cytosolic	R291P	>10 000	0	0.04
Cytosolic	R311G	Enzyme inactive	‐	‐
Cytosolic	R311L	>10 000	14	0.35
Cytosolic	R311P	>10 000	0	0.16
Cytosolic	R311Q	>10 000	1.9	0.46
Cytosolic	R332G	Enzyme inactive	‐	‐
Cytosolic	R332S	Enzyme inactive	‐	‐
Cytosolic	R332K	Enzyme inactive	‐	‐
Cytosolic	R332M	Enzyme inactive	‐	‐
Cytosolic	R332T	Enzyme inactive	‐	‐
Cytosolic	R332W	Enzyme inactive	‐	‐

Mutations were introduced into the cytosolic GS1.1 isoform and purified enzymes were assayed *in vitro*. IC₅₀ values represent the inhibitor concentration required to reduce activity by 50%, calculated from dose–response curves. “Inhibition at 10^−2^ M” indicates the percentage inhibition at saturating glufosinate‐ammonium relative to uninhibited control. ‘Remaining activity’ represents the baseline enzymatic activity in the absence of inhibitor, expressed as a percentage of wild‐type (WT) activity. Variants designated ‘inactive’ exhibited no detectable activity under assay conditions and IC₅₀ values are not reported. Bold values indicate the wild‐type (WT) enzyme used as the reference for comparison.

Overall, several mutations in cytosolic GS1.1 catalytic domain dramatically reduce sensitivity to GFA but impair catalytic efficiency simultaneously. This indicates that resistance‐conferring substitutions come at a substantial cost to enzyme functionality.

A panel of additional GS1.1 variants carrying putative target‐site resistance mutations in the GS1.1 subunits interaction domain, where GFA is predicted to bind, were obtained by modeling simulation (Fig. S2) and evaluated for both baseline catalytic activity (expressed as % of wild‐type GS1.1) and resistance index (RI) to GFA (Fig. 6 and Table 3). Around 10 of the tested variants clustered near the origin, having low RIs (<5) and reduced activity, often below 40% of the wild‐type level, indicating that these substitutions were either functionally neutral in terms of resistance or detrimental to enzyme performance. A smaller subset displayed moderate resistance (RI 8–12) but at the cost of substantially reduced catalytic activity (<40%). Four variants had very high GS activity ranging from 200% to 800% of the wild‐type enzyme but also low RI. Finally, another 10 variants display mild and high RI (7 to 750) but severe reduction in enzyme activity below 20%. To further explore the relevance of previously reported target‐site alterations, the *Eleusine indica* GS1.1 S59G mutation was also evaluated in this study for its effects on catalytic efficiency and inhibitor sensitivity.

**Table 3:** Additional mutations introduced into GS1.1 isoform of *Amaranthus palmeri*, obtained by modeling prediction

GS1.1 isoform	Mutation	Remaining activity [%]	Glufosinate‐ammonium IC_50_[μM] *P* < 0.0001	Resistance index (RI)
Cytosolic	**WT**	**100**	**21.7**	**1**
Cytosolic	R34A	10	182	8.4
Cytosolic	K36A	14	248	11.5
Cytosolic	R38E	166	4.8	0.2
Cytosolic	Y55L	37	17	0.8
Cytosolic	D56C	5	3.9	0.2
Cytosolic	D56S	2	56	2.6
Cytosolic	S58M	105	94	4.3
Cytosolic	S59N	4	39.5	1.8
Cytosolic	T60Q	5	366	16.9
Cytosolic	E69L	39	246	11.4
Cytosolic	G155F	31	254	11.7
Cytosolic	Y158A	5	1401	64.6
Cytosolic	Y158T	29	76	3.5
Cytosolic	C159H	1	4046	186.5
Cytosolic	G160T	13	405	18.7
Cytosolic	V161N	6	64	3.0
Cytosolic	V161S	23	39	1.8
Cytosolic	G162N	2	489	22.6
Cytosolic	A163Y	101	54	2.5
Cytosolic	K165S	71	7.6	0.3
Cytosolic	K165V	209	33	1.5
Cytosolic	S174E	529	1	0.0
Cytosolic	K177L	823	1.3	0.1
Cytosolic	V193T	85	69	3.2
Cytosolic	G245P	22	247	11.4
Cytosolic	E297A	9	6.9	0.3
Cytosolic	E297Q	13	0.6	0.0
Cytosolic	E297S	19	0	0.0
Cytosolic	A309L	34	204	9.4
Cytosolic	A309Y	4	17 355	799.7
Cytosolic	R316A	6	313	14.4
Cytosolic	R316E	2	197	9.1

IC₅₀ values represent the inhibitor concentration required to reduce activity by 50%, calculated from dose–response curves. Remaining activity represents the baseline enzymatic activity in the absence of inhibitor, expressed as a percentage of wild‐type (WT) activity.

### 3.7 Assessing GS1.1 S59G response to GFA

**Figure 5:**
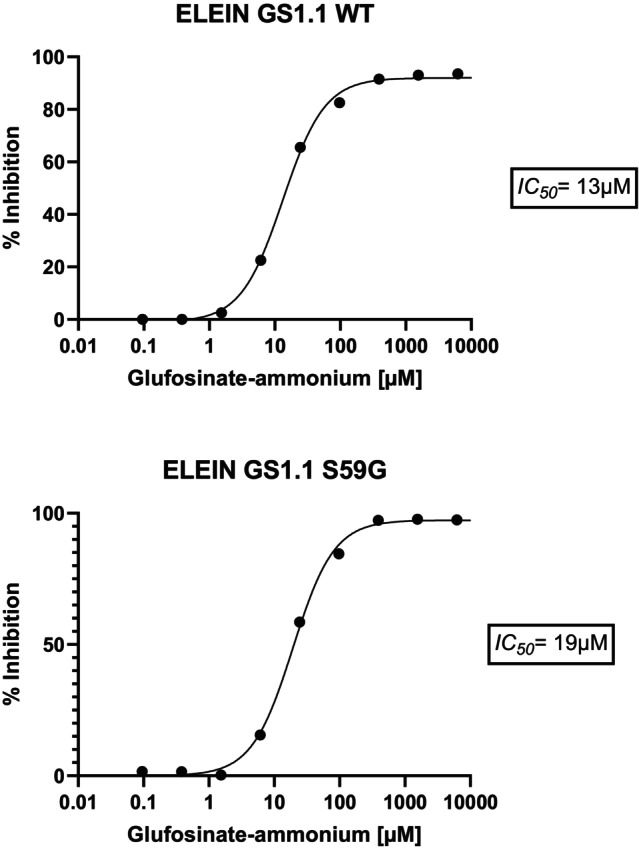
Inhibition of *Eleusine indica* GS1.1 wild‐type (WT) and S59G mutant enzymes by GFA. Enzyme inhibition was measured as the percentage decrease in activity relative to uninhibited control across a range of GFA concentrations. Dose–response curves were fitted using a four‐parameter logistic model. The WT enzyme (upper panel) exhibited an IC₅₀ of 13 μM, while the S59G mutant (lower panel) had an IC₅₀ of 19 μM. Data points represent the mean ± standard error of three replicate measurements.

To assess whether the S59G amino acid substitution in ELEIN GS1.1 affects sensitivity to GFA, as previously published,^24^ inhibition kinetics were compared between the wild‐type and mutant enzymes. Both variants displayed highly similar dose–response profiles, with enzymatic activity progressively declining as GFA concentration increased. The IC₅₀ of GFA on wild‐type and S59G variant enzymes were 13 and 19 μM, respectively, representing only a minor, biologically insignificant difference with regards to the resistance mechanism (Fig. 5). Maximal inhibition was achieved at similar concentrations in both cases, and the shape of the inhibition curves was comparable, indicating no detectable change in inhibitor binding or susceptibility. However, the S59G substitution results in a higher enzyme activity compared to the wild‐type enzyme (Fig. S3), suggesting that the S59G substitution does not alter GS1.1 function with respect to GFA sensitivity, but it increases the overall catalytic activity of the enzyme.

## 4 DISCUSSION

Copy number, transcript and sequence analyses of GS genes in the *A. palmeri* CCR population revealed a resistance mechanism distinct from that of previously characterized GFA‐resistant populations.^17^, ^28^ While resistance was attributed to increased copy number and expression of GS2 genes in the past, the CCR population had minimal copy number variation. Most CCR individuals had GS copy numbers comparable to a susceptible reference (0.8–1.5‐fold), with only a few plants displaying moderate increases in GS2.2 (up to ~2.0‐fold) and GS2.1 (Fig. 1(A)). GS CN variation was not prevalent among the assessed plants and did not exceed thresholds typically associated with gene amplification‐driven resistance. This contrast shows the genetic variability of glufosinate resistance mechanisms across populations and highlights the CCR population's unique profile lacking substantial GS amplification. There was no difference in GS expression between the resistant populations and the susceptible reference (Fig. 1(B)).

The G255D substitution was identified in GS2.2 in heterozygous form in 18 out of 23 CCR untreated plants. This residue is located adjacent to E251, a known GFA binding residue according to structural modeling.^29^ The substitution from glycine to aspartic acid introduces a charged residue in a conserved domain of the enzyme, which altered the active site conformation and reduced catalytic efficiency. There was a 42% decrease in GS activity for the G255D variant compared to wild‐type GS2.2 (Fig. 2). In addition, GFA inhibition assays demonstrated that while the wild‐type enzyme exhibited a typical dose‐dependent inhibition curve (IC₅₀ = 1.1 × 10^−4^ M), the G255D variant showed no measurable inhibition across the tested concentrations, indicating complete insensitivity to GFA at the enzymatic level (Fig. 3).

However, when the G255D variant was ectopically expressed in *Arabidopsis thaliana*, it failed to confer GFA tolerance *in planta*. Transgenic lines expressing G255D exhibited similar injury symptoms and biomass reduction as wild‐type plants across all GFA treatment rates (Fig. 4(A)). A parallel set of transgenics expressing the corresponding mutation in the *Arabidopsis* GS2.2 backbone (G254D) also resulted in a sensitive phenotype (Fig. 4(B)). Although G255D confers resistance to GFA at the enzyme level, it did not confer resistance at the plant level under the conditions tested. The discrepancy between *in vitro* and *in vivo* results in *Arabidopsis* likely reflects the complex, multi‐level regulation of GS and substantial functional and spatial redundancy among GS isoforms.^30^ Moreover, heterologous expression may fail to fully integrate into native regulatory or metabolic contexts, such as appropriate expression level, subcellular environment, or buffering within nitrogen assimilation pathways.^31^ CRISPR/Cas9‐mediated editing of *A. palmeri* GS could overcome these limitations and enable direct testing of the role of G255D in GFA resistance. Overall, these observations emphasize the need for cautious interpretation of *in vitro* experiments when evaluating potential resistance‐conferring mutations. It remains to be determined whether this substitution represents a background polymorphism, an early‐stage adaptation, or a mutation that requires co‐occurring mechanisms to manifest a resistant phenotype. The absence of G255D among GFA‐surviving plants could also suggest that this mutation may be detrimental to GFA tolerance. Overall, the resistance mechanism of the CCR population is unlikely to be based on target‐site mutations or target overexpression.

Further insights into GS structure–function relationships can contextualize these findings. Glufosinate acts as a competitive inhibitor of glutamate, binding at the same site due to structural similarity with the intermediate γ‐glutamyl phosphate. Key residues involved in GFA binding, including E131, E192, G245, H249, R291, and R311, are conserved across GS isoforms.^29^, ^32^ The G255D mutation lies in proximity to these residues and may influence local conformational dynamics affecting both substrate and inhibitor interaction.

**Figure 6:**
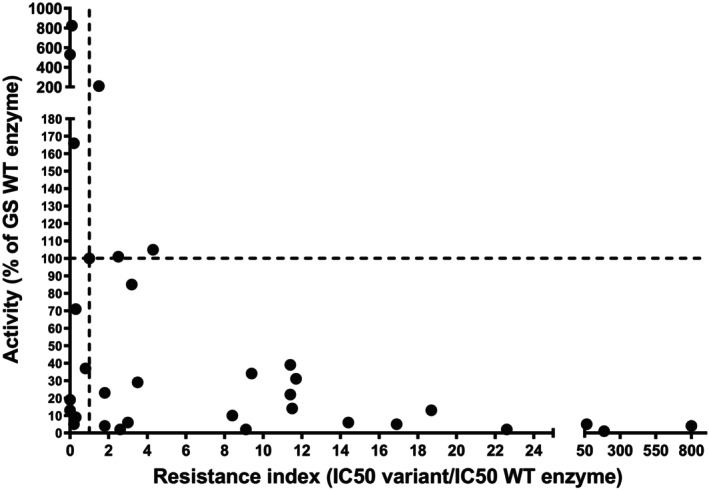
Relationship between resistance index (RI) and enzyme activity of *Amaranthus palmeri* cytosolic GS1.1 variants.

To explore this further, different codon variants were introduced at key GS1.1 residues to evaluate their effects on GFA sensitivity and catalytic function. Inclusion of multiple substitutions at single positions like E131A/D/G/K, E192A/D/G/K/Q/V, G245N/R/V, H249L/N/Q/P/Y, R311G/L/P/Q, R322G/S/K/M/T/V (Table S3) allowed assessment of whether loss of herbicide sensitivity was a general outcome of amino acid replacement at these sites or specific to particular residue chemistries. The results indicate that although many of these variants resulted in loss of GFA sensitivity, they also reduced catalytic activity to negligible levels, which limits the potential of such substitutions to confer resistance at the plant level (Table 2). Additionally, variants obtained by molecular prediction method rendered similar results. Variants retaining more than 65% of wild‐type catalytic activity and exhibiting a RI substantially higher than that of the GS2.2 G255D reference would warrant further evaluation for resistance; however, molecular docking analyses did not identify substitutions meeting these criteria (Fig. 6, Table S3). It is to be noted that while G255D was found in GS2.2, the panel of target site mutants was tested using the GS1.1 isoform backbone. However, since GS1.1 and GS2.2 share a high sequence similarity, especially high conservation of the residues involved in GFA binding,^29^ it would be expected that target‐site mutations tested in GS1.1 are likely to produce the same effect in GS2.2. The panel of GS1.1 target site mutations were performed prior to the detection of GS2.2 G255D.

In a study involving soybean cell suspension culture, resistant lines displayed a 4.6‐fold higher IC₅₀ of GS activity relative to untreated controls, indicating reduced sensitivity of the enzyme to inhibition. Sequencing revealed multiple nucleotide substitutions in the GS gene, including a H249Y change within the substrate/inhibitor binding region, consistent with altered target‐site interaction.^25^ Although no enzyme stability assays were performed, the authors proposed that these substitutions may reduce enzyme stability while simultaneously diminishing herbicide‐binding. Such mutations can be recovered under *in vitro* conditions, where minimal metabolic demands permit survival of impaired cells, but are unlikely to persist in field populations due to associated fitness costs. In contrast, DNA shuffling of rice GS1 produced a variant carrying the R295K substitution that conferred measurable GFA resistance when expressed in *Arabidopsis*.^26^ Although the level of resistance was weaker than that conferred by *bar/pat*, this study demonstrates the feasibility of engineering GS‐based tolerance through specific amino acid substitutions. A study in *L. multiflorum* identified a D173N substitution in the GS2 gene that was proposed to confer GFA resistance.^33^ However, this hypothesis was disputed in a subsequent study reporting no difference in GS activity between resistant and susceptible biotypes.^23^ Structural modeling further demonstrated that residue 173 is distant from the catalytic site and unlikely to influence herbicide binding. All these studies illustrate the structural constraints of GS: some substitutions disrupt the herbicide binding but impair catalytic function, while others shift herbicide sensitivity without major loss of activity, emphasizing why GS mutations rarely translate into field‐relevant resistance.

The GFA‐resistant *E. indica* population originally described by Jalaludin *et al*. (2015)^10^ as exhibiting up to 20‐fold resistance was subsequently investigated by Zhang *et al*. (2021).^24^ In that study, the S59G substitution in *EiGS1‐1* was identified and functionally tested. In the present study, inhibition kinetics of purified recombinant GS1.1 revealed no meaningful difference in glufosinate IC₅₀ between the wild‐type and S59G variants. Differences between studies may reflect methodological variation, including enzyme source, expression system, protein purification strategy, assay buffer composition, substrate concentrations, or inhibitor exposure conditions, all of which can influence apparent IC₅₀ estimates. Notably, the S59G variant displayed significantly higher basal catalytic activity than the wild‐type enzyme (Fig. S3; *P* < 0.05), suggesting that increased GS activity, may contribute to tolerance under certain physiological exposure scenarios.

Overall, this study constitutes a critical reference point for evaluating the functional consequences of GS mutations in GFA resistance research. In addition, the absence of GS gene amplification or overexpression, the inability of identified GS substitutions to confer GFA tolerance *in planta*, and the lack of G255D among GFA survivors indicates that TSR is unlikely to explain the CCR resistant phenotype. Instead, these findings strongly suggest that NTSR mechanisms, such as altered herbicide uptake, translocation, sequestration, or enhanced metabolism, are the most plausible contributors to GFA resistance in this population.

## 5 CONCLUSION

GFA resistance in the CCR *A. palmeri* population is not explained by GS amplification or the GS2.2 G255D substitution. While G255D abolished GFA inhibition *in vitro*, its reduced catalytic efficiency and failure to confer tolerance *in planta* highlight the strong functional constraints on target‐site mutations. Consistent with this, broader mutational analyses confirmed that most substitutions disrupting GFA binding also compromise enzyme activity, underscoring the limited adaptive potential of GS alterations. These findings emphasize that GFA resistance in field populations is unlikely to arise from target‐site modifications alone, reinforcing the need to investigate alternative, non–target‐site mechanisms.

## CONFLICT OF INTEREST

The authors declare no conflicts of interest.

## Supporting information


**Figure S1.** Glutamine synthetase 1 protein sequence of *Zea mays* (GS1.1) and *Amaranthus palmeri*. In blue squares are depicted the residues involved in P‐PPT (phosphinothricin or glufosinate) as reported by Unno *et al*., 2006.
**Figure S2.** Glutamine synthetase 1 protein sequence of *Amaranthus palmeri*. In blue squares are depicted the residues involved in P‐PPT (phosphinothricin or glufosinate) as obtained by docking simulation (see material and methods).
**Figure S3.** Enzyme activity of cytosolic glutamine synthetase 1 (GS1.1) from Eleusine indica. Activities of the wild‐type (WT) and the S59G variant were determined by measuring the rate of absorbance change (mOD min^−1^) *in vitro*. Bars represent mean values ± SE of three independent enzyme preparations (*n* = 3). Statistical significance was assessed using a two‐tailed Student's *t*‐test (*P* < 0.05).
**Table S1.** Primer information for copy number and expression analysis.
**Table S2.** Primer information for cDNA sequencing.
**Table S3.** Possible amino acid substitutions in cytosolic *Amaranthus palmeri* GS1.1 resulting from single nucleotide polymorphisms (SNPs). The table lists residues in the GS1 protein known to interact with glufosinate (Unno *et al*., 2006). Each residue is followed by the set of alternative amino acids that could result from a SNP, based on one single base changes.
**Table S4.** GS2.2 sequencing of glufosinate‐ammonium survivors from the CCR population. The GS2.2 coding region was sequenced in *Amaranthus palmeri* plants that survived a single application of 1X glufosinate‐ammonium. All analyzed survivors (*n* = 15) carried the wild‐type glycine residue at position 255 (G255), and the G255D substitution was not detected in any surviving individual. GS2.2 sequences were obtained by Sanger sequencing.

## Data Availability

The data that support the findings of this study are available on request from the corresponding author. The data are not publicly available due to privacy or ethical restrictions.
