# Supplementary material for: Analysis of glutamine synthetase target‐site mutations and their role in endowing glufosinate‐ammonium resistance
Source: Pest Manag Sci. 2026 May 11;82(8):7633–44. doi: 10.1002/ps.70827 (PMC13352219; doi:10.1002/ps.70827)
Supplement: Supplementary file 1 — Figure S1. Glutamine synthetase 1 protein sequence of Zea mays (GS1.1) and Amaranthus palmeri. In blue squares are depicted the residues involved in P‐PPT (phosphinothricin or glufosinate) as reported by Unno et al., 2006. Figure S2. Glutamine synthetase 1 protein sequence of Amaranthus palmeri. In blue squares are depicted the residues involved in P‐PPT (phosphinothricin or glufosinate) as obtained by docking simulation (see material and methods). Figure S3. Enzyme activity of cytosolic glutamine synthetase 1 (GS1.1) from Eleusine indica. Activities of the wild‐type (WT) and the S59G variant were determined by measuring the rate of absorbance change (mOD min−1) in vitro. Bars represent mean values ± SE of three independent enzyme preparations (n = 3). Statistical significance was assessed using a two‐tailed Student's t‐test (P < 0.05). Table S1. Primer information for copy number and expression analysis. Table S2. Primer information for cDNA sequencing. Table S3. Possible amino acid substitutions in cytosolic Amaranthus palmeri GS1.1 resulting from single nucleotide polymorphisms (SNPs). The table lists residues in the GS1 protein known to interact with glufosinate (Unno et al., 2006). Each residue is followed by the set of alternative amino acids that could result from a SNP, based on one single base changes. Table S4. GS2.2 sequencing of glufosinate‐ammonium survivors from the CCR population. The GS2.2 coding region was sequenced in Amaranthus palmeri plants that survived a single application of 1X glufosinate‐ammonium. All analyzed survivors (n = 15) carried the wild‐type glycine residue at position 255 (G255), and the G255D substitution was not detected in any surviving individual. GS2.2 sequences were obtained by Sanger sequencing. [file PS-82-7633-s001.docx]

**Figure S1. Glutamine synthetase 1 protein sequence of *Zea mays* (GS1.1) and *Amaranthus palmeri*.** In blue squares are depicted the residues involved in P-PPT (phosphinothricin or glufosinate) as reported by Unno *et al*., 2006.

Cytosolic *Zea mays* GS1.1


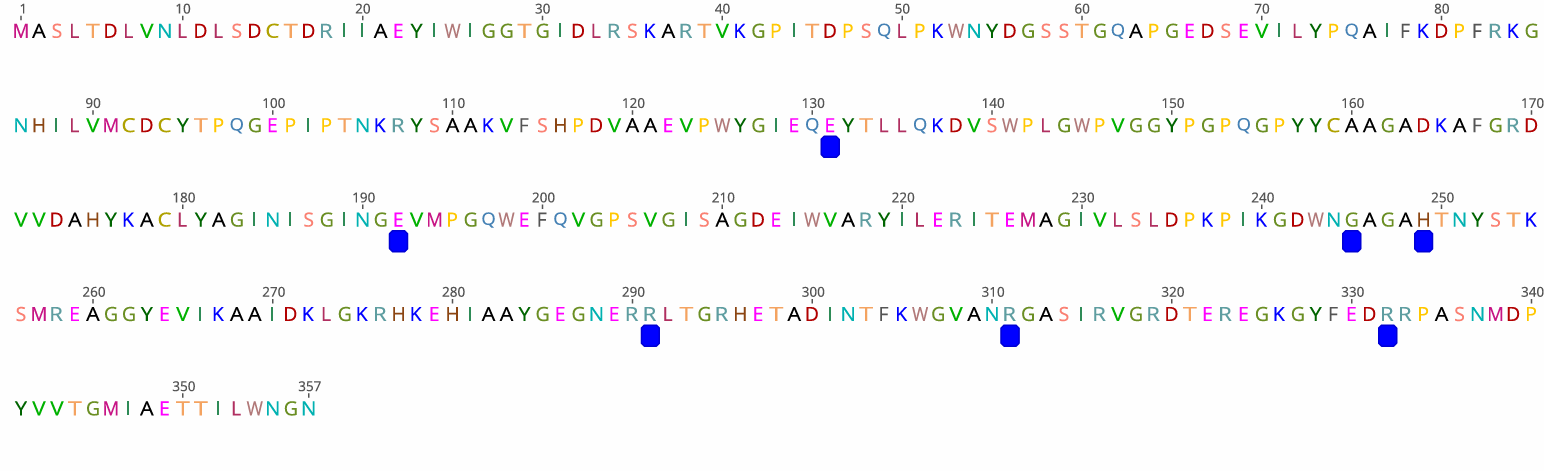


Cytosolic *Amaranthus palmeri* GS1.1


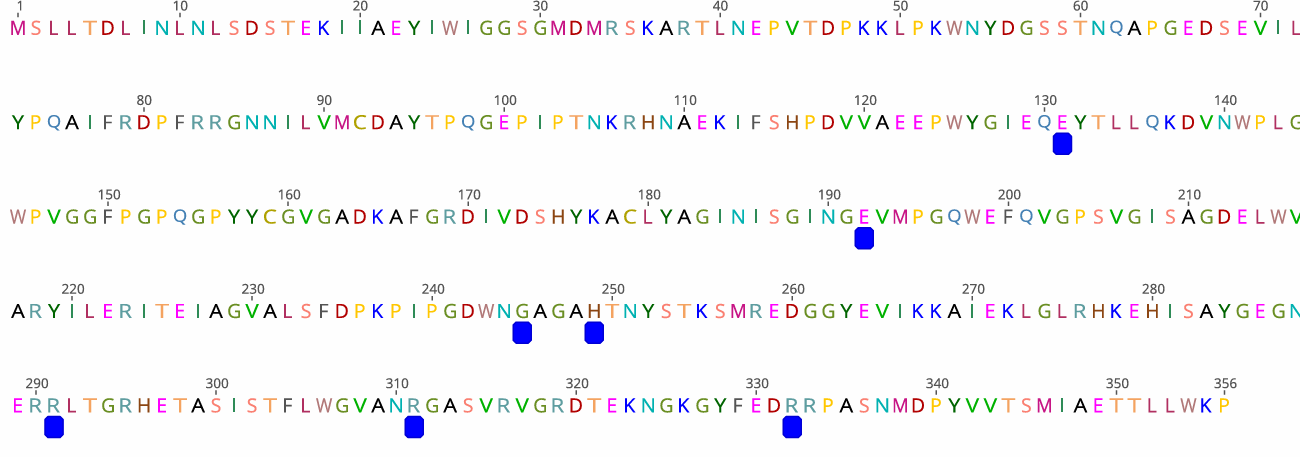


**Figure S2. Glutamine synthetase 1 protein sequence of *Amaranthus* *palmeri*.** In blue squares are depicted the residues involved in P-PPT (phosphinothricin or glufosinate) as obtained by docking simulation (see material and methods).

Cytosolic *Amaranthus palmeri* GS1.1

**
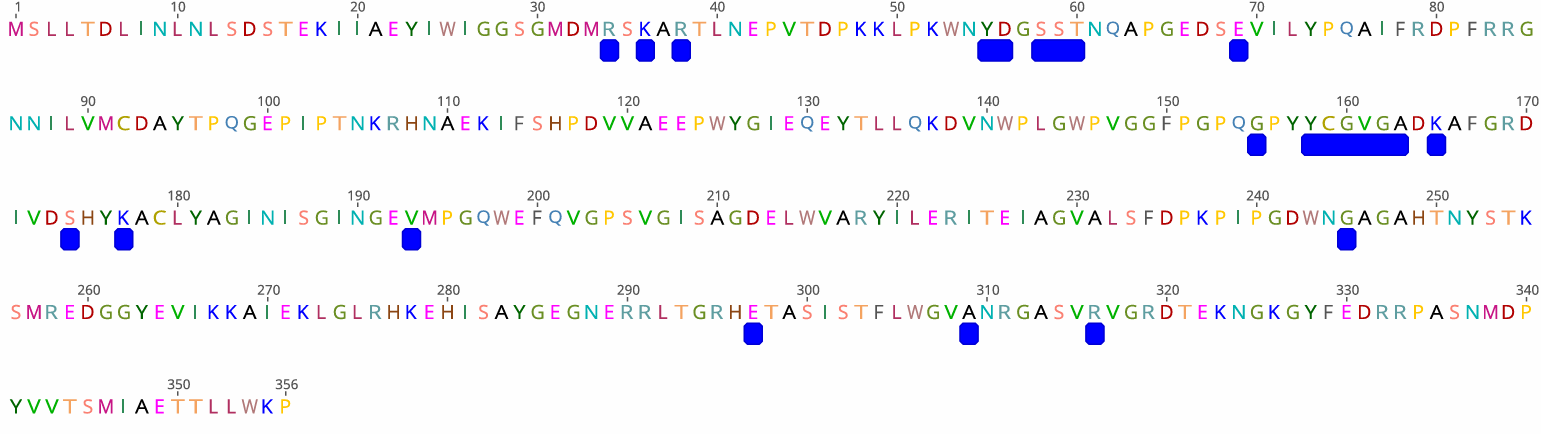
**

**Figure S3. Enzyme activity of cytosolic glutamine synthetase 1 (GS1.1) from Eleusine indica.** Activities of the wild-type (WT) and the S59G variant were determined by measuring the rate of absorbance change (mOD min⁻¹) in vitro. Bars represent mean values ± SE of three independent enzyme preparations (n=3). Statistical significance was assessed using a two-tailed Student’s t-test (*P* < 0.05).

**Table S1.** Primer information for copy number and expression analysis

| **PRIMER NAME** | **SEQUENCE INFORMATION** | **METHOD INFORMATION** | |
| --- | --- | --- | --- |
| GS 1.1 CNV for | TGTGTGATGCCTATACTCCACA |  | dPCR CNV |
| GS 1.1 CNV rev | TACCATGGTTCCTCGGCAAC |  | dPCR CNV |
| GS 1.1 CNV probe | AGGAGAGCCAATCCCAACCAACA | Fam / BMN-Q535 | dPCR CNV |
| GS 1.2 CNV for | TCGATGAGGAATGATGGTGGGATCGA |  | dPCR CNV |
| GS 1.2 CNV rev | ACCAGTGAGTCTTCTCTCGTTGCCC |  | dPCR CNV |
| GS 1.2 CNV probe | AGCGATTGAGAAGTTGAGCCTCCGCCACAA | Hex / BMN-Q535 | dPCR CNV |
| GS 2.1 CNV for | CTCTATCCGTGTGGGTCGTG |  | dPCR CNV |
| GS 2.1 CNV rev | ACACTCACATACCCAGGATA |  | dPCR CNV |
| GS 2.1 CNV probe | GGCCTGCCTCAAACATGGACCC | ROX / BMN-Q620 | dPCR CNV |
| GS 2.2 CNV for | TGGTAACAGGTTTGCTCGCCGA |  | dPCR CNV |
| GS 2.2 CNV rev | TGGTTGGAATTACACATTAAGAGCGAGT |  | dPCR CNV |
| GS 2.2 CNV probe | CCCACACTTGAGGCCGAGTCACTTGCAGC | TAMRA / BMN-Q2 | dPCR CNV |
| Actin CNV for | GCGGAAAGCTAAGCGTGAAC |  | dPCR CNV |
| Actin CNV rev | CAGACCTGCTCTGGAGTCAG |  | dPCR CNV |
| Actin CNV probe | ACTGGAGGAAAAGGCGGATGCTGCA | Cy5/ BMN-Q620 | dPCR CNV |
| GS 1.1 Expression for | CCATTCAGGAGGGGCAACAA |  | dPCR Expression |
| GS 1.1 Expression rev | TGCAGCAAGGTGTACTCCTG |  | dPCR Expression |
| GS 1.1 Expression probe | TGCCGAGGAACCATGGTACGGT | Fam / BMN-Q535 | dPCR Expression |
| GS 1.2 Expression for | TGCTGAAATTGCTGGAGCCA |  | dPCR Expression |
| GS 1.2 Expression rev | GCGGAGGCTCAACTTCTCAA |  | dPCR Expression |
| GS 1.2 Expression probe | AGGGTGACTGGAATGGTGCTGGT | Hex / BMN-Q535 | dPCR Expression |
| GS 2.1 Expression for | CTCTATCCGTGTGGGTCGTG |  | dPCR Expression |
| GS 2.1 Expression rev | TGCGATACACGATTTATGATTGAA |  | dPCR Expression |
| GS 2.1 Expression probe | GGCCTGCCTCAAACATGGACCC | ROX / BMN-Q620 | dPCR Expression |
| GS 2.2 Expression for | CACGGAAAAGGCAGGCAAAG |  | dPCR Expression |
| GS 2.2 Expression rev | TTCTGGTTCAATTAATGGTTGGAA |  | dPCR Expression |
| GS 2.2 Expression probe | AGGCCGAGTCACTTGCAGCT | TAMRA / BMN-Q2 | dPCR Expression |
| Actin Expression for | AAGACTACTGCTGAAGGCCG |  | dPCR Expression |
| Actin Expression rev | TCCTCTAGTTGGTTTTGCTTCTCT |  | dPCR Expression |
| Actin Expression probe | AGCGAGCTAGACAGAAACGTGCT | Cy5/ BMN-Q620 | dPCR Expression |

**Table S2.** Primer information for cDNA sequencing

| **PRIMER NAME** | **SEQUENCE INFORMATION** |
| --- | --- |
| GS 1.1 for 01 | AGAACATACTCATCTTCCACTTCTC |
| GS 1.1 rev 01 | CCAGCAGAGATTCCAACAGACGGGCCG |
| GS 1.1 for 02 | TGGCCCCTTGGTTGGCCTGTAGG |
| GS 1.1 rev 02 | TTGATTAAGTTCGTGGCCGC |
| GS 1.2 for 01 | GGATCGGAGGATCTGGCTTG |
| GS 1.2 rev 01 | CCACTGTCCGGGCATAACTT |
| GS 1.2 for 02 | TGGCTTCCCAGGTCCTCAGGGG |
| GS 1.2 rev 02 | CAAGAAATTCCAAATTCACATTAACATRAYC |
| GS 2.1 for 01 | CGACACCCTTTTCCGATCA |
| GS 2.1 rev 01 | AACACTTGGGCCAACTTGGA |
| GS 2.1 for 02 | TGGGATGGCCAGTGGGAGCC |
| GS 2.1 rev 02 | ACACTCACATACCCAGGATA |
| GS 2.2 for 01 | TCACTCTCTCTCTAGTTACTTSACGCC |
| GS 2.2 rev 01 | GGCTCCCACTGGCCATCCCAAAGGCCA |
| GS 2.2 for 02 | GTTGAGCACCCATCTGAGCT |
| GS 2.2 rev 02 | TCCCTCGTTCAAGAATATGCGA |

**Table S3. Possible amino acid substitutions in cytosolic Amaranthus palmeri GS1.1 resulting from single nucleotide polymorphisms (SNPs).** The table lists residues in the GS1 protein known to interact with glufosinate (Unno *et al*., 2006). Each residue is followed by the set of alternative amino acids that could result from a SNP, based on one single base changes.

| **Cytosolic AMAPA GS1.1** | |
| --- | --- |
| **Residue** | **Possible substitution deriving from a SNP** |
| E131 | A |
|  | D |
|  | G |
|  | K |
|  | Q |
|  | V |
| E192 | A |
|  | D |
|  | G |
|  | K |
|  | Q |
|  | V |
| G245 | A |
|  | C |
|  | N |
|  | R |
|  | S |
|  | V |
| H249 | L |
|  | N |
|  | P |
|  | Q |
|  | R |
|  | Y |
| R291 | C |
|  | G |
|  | H |
|  | L |
|  | P |
|  | S |
| R311 | G |
|  | L |
|  | P |
|  | Q |
| R332 | G |
|  | K |
|  | M |
|  | S |
|  | T |
|  | W |

| **CCR population** | **GS2.2** |
| --- | --- |
| Plant 1 | G255 |
| Plant 2 | G255 |
| Plant 3 | G255 |
| Plant 4 | G255 |
| Plant 5 | G255 |
| Plant 6 | G255 |
| Plant 7 | G255 |
| Plant 8 | G255 |
| Plant 9 | G255 |
| Plant 10 | G255 |
| Plant 11 | G255 |
| Plant 12 | G255 |
| Plant 13 | G255 |
| Plant 14 | G255 |
| Plant 15 | G255 |

**Table S4. GS2.2 sequencing of glufosinate-ammonium survivors from the CCR population.** The GS2.2 coding region was sequenced in Amaranthus palmeri plants that survived a single application of 1X glufosinate-ammonium. All analyzed survivors (n = 15) carried the wild-type glycine residue at position 255 (G255), and the G255D substitution was not detected in any surviving individual. GS2.2 sequences were obtained by Sanger sequencing.
